# Complete Genome Sequence of a Chikungunya Virus Imported from Bali to Germany

**DOI:** 10.1128/genomeA.00164-15

**Published:** 2015-03-26

**Authors:** Silke Wölfel, Patrick Vollmar, David Poluda, Sabine Zange, Markus H. Antwerpen, Thomas Löscher, Gerhard Dobler

**Affiliations:** a Bundeswehr Institute of Microbiology, Munich, Germany; b Department of Infectious Diseases and Tropical Medicine, University of Munich, Munich, Germany; c DZIF German Centre for Infection Research, Ludwig Maximilians University of Munich, Munich, Germany

## Abstract

Chikungunya virus (CHIKV) strain DH130003 was isolated from a traveler with Chikungunya fever returning from Bali to Germany. Although strains of the east-central/south African lineage bearing the A226V mutation have predominated in most parts of Asia since 2005, CHIKV DH130003 belongs to the Asian lineage.

## GENOME ANNOUNCEMENT

Chikungunya virus (CHIKV) is a member of the genus *Alphavirus* within the family *Togaviridae* and is transmitted by Aedes mosquitoes. Chikungunya fever is a cause of fever and arthralgia in inhabitants and travelers from Africa and Asia and, recently, also from the Americas (1, 2). On the Indonesian island of Bali, only sporadic outbreaks have been reported so far (3). In December 2012, a 53-year-old female traveler developed Chikungunya fever with headache, arthralgia, and exanthema in combination with lymphadenopathy and mild hepatitis 3 days after returning from an 18-day trip exclusively to Bali. The virus was isolated by cell culture. Whole-genome sequencing was carried out after extracting RNA from original patient serum and cell culture supernatant using a QIAamp viral RNA minikit (Qiagen, Hilden, Germany) and reverse transcription by superscript III reverse transcriptase (Life Technologies, Darmstadt, Germany). The 3′ untranslated region (UTR) was sequenced using a method previously described (4), the 5′ UTR was analyzed by using a 5′ rapid amplification of cDNA ends kit (Invitrogen) according to the manufacturer’s recommendations. Sequencing was carried out by GATC Biotech (Konstanz, Germany) using Sanger sequencing. Assembly of nucleic acid and protein sequences was performed using BioEdit (version 7.1.11). The complete genome sequence was determined to be 11,979 nucleotides (nt). Two open reading frames (ORFs) flanked by the 5′ UTR and 3′ UTR were identified. The ORFs contained the nonstructural polyprotein (7,401 nt; 2,467 amino acids [aa]) with an opal read-through site at the C-terminal region of the nonstructural protein (NSP) 3 and the structural polyprotein (3,744 nt; 1,248 aa). The nonstructural polyprotein consisted of the NSP1, NSP2, NSP3, and NSP4. Within NSP3, a 7-aa deletion was observed which has only been found in a few Malaysian CHIKV strains and one strain from New Caledonia (GenBank accession no. HE806461.1) so far. The structural polyprotein consisted of a capsid protein, of the envelope protein 3 (E3), E2, 6K, and E1. The A226V mutation, which increases the susceptibility of Aedes albopictus to CHIKV, was not detected in the envelope E1 protein sequence (5). In the 3′ UTR, a 28-nt deletion was observed which had only been reported in the strain from New Caledonia but not in the Malaysian strains. Since the beginning of the Indian Ocean islands outbreaks, the pandemic ECSA strain containing the A226V mutation has been spreading throughout Asia and one strain has been found in Indonesia (GenBank accession no. KC862329.1). Although it appears to replace the old Asian lineage that has been endemic in Asia for years (6), our findings suggest that it has not yet shown the capability of replacing the currently circulating strains of the Asian lineage in Indonesia. To what extent the deletion of 7 amino acids in the NSP3 or the deletion of 28 nucleotides in the 3′ UTR may have contributed to the persistence of this strain of the Asian lineage remains unclear.

### Nucleotide sequence accession number.

The complete genome sequence of CHIKV strain DH130003 has been deposited in GenBank with the accession no. KM673291.
